# The effect of hyperbaric oxygen therapy on hematological indices and biochemical parameters in patients with diabetic foot

**DOI:** 10.1097/MD.0000000000037493

**Published:** 2024-03-22

**Authors:** Erdinç Ercan, Gamze Aydin, Bülent Erdoğan, Fatih Özçelik

**Affiliations:** a Department of Aerospace Medicine, University of Health Sciences, Ankara, Türkiye; b Clinic of Nephrology, Gulhane Training and Research Hospital, Ankara, Türkiye; c Department of Medical Biochemistry, University of Health Sciences, İstanbul, Türkiye.

## Abstract

**Background:**

Diabetes Mellitus (DM) is a metabolic disease with a high morbidity and mortality and increasing in prevalence all over the world. Due to the hypoxic, ischemic, inflammatory, and infective environment in DM, diabetic foot ulcers have been treated with medico-surgical interventions and adjuvant hyperbaric oxygen Therapy (HBOT). The purpose of this study was to evaluate the effects of HBOT on hematological indices and biochemical parameters in patients with diabetic foot.

**Methods:**

The study group was formed from the file records of 103 male patients who applied to Yunus Emre State Hospital HBOT Center between September 1, 2016 and December 31, 2020, and were treated HBOT with a multidisciplinary approach.

**Results:**

There were negative low correlations between number of HBOT sessions and Mean Corpuscular Hemoglobin (MCH) (*P* = .037, r = −0.207) and Blood Urea Nitrogen (BUN) (*P* = .037, r = −0.222). White Blood Cell Count (WBC), Neutrophils (NEU), Monocytes (MON), Platelet Count (PLT), and Plateletcrit (PTC) parameters were found to be decreased, and an increase in lymphocytes (LYM), Eosinophils (EOS), Mean Corpuscular Hemoglobin Concentration (MCHC), and Red Cell Distribution Width (RDW) parameters were detected after the treatments (*P* < .05). Again, after the treatment, glucose (Glu), C-Reactive Protein (CRP), direct bilirubin, and total protein (TP) levels were decreased, and uric acid (UA) levels increased (*P* < .05).

**Conclusion:**

HBOT improved hematological indices in patients and had a beneficial effect on biochemical parameters, particularly Glu and CRP levels. Adjuvant HBOT alleviates diabetic inflammation and has a beneficial effect on diabetic patient treatment.

## 1. Introduction

Diabetic foot is the most common complication of diabetes caused by the combined effect of diabetes-related vascular disease and neuropathy. In the pathogenesis, trauma, or an increase in pressure load on foot, leads to portal of entry for infections.^[1]^ International Diabetes Federation published Diabetes Atlas in 2019 and in this report the global diabetic foot incidence was 2% (9.26 million) and approximately 1% of diabetic patients suffer from lower extremity amputation at some point in their lives. In addition, the recurrence rates of diabetic foot ulcers have been reported as 40% within 1 year and 65% within 5 years.^[2]^ Diabetic foot is the most common cause of non-traumatic foot amputations. The number of diabetic foot amputations performed yearly in Türkiye is approximately twelve thousand.^[3]^ Diabetic foot causes deterioration in the quality of life, loss of workday, and psychological problems, as well as increases hospitalization, prolongs hospital stay and increases expenses.^[4]^ Diabetes Mellitus (DM) interferes with the metabolism of cells and tissues and wound healing is impaired.^[5]^

HBOT performed in a chamber higher pressure than ambient (atmospheric) pressure by breathing 100% oxygen through a mask, endotracheal tube, or hood. HBOT increases the dissolved oxygen in the blood and creates a higher-pressure gradient suitable for the diffusion of oxygen into complicated tissues. In hypoxic tissues, the increase in oxygen tensions has many effects that can promote wound healing. HBOT can increase angiogenesis and fibroblast proliferation by increasing expression of the vascular endothelial growth factor and the fibroblast growth factor. Also, hyperoxia leads to vasoconstriction that can reduce tissue edema. HBOT increases the bacterial-killing activity of leukocytes and suppresses the inflammation via reducing the expression of pro-inflammatory cytokines.^[6–8]^ Because of these positive effects, HBOT has been used as an adjuvant treatment for diabetic foot ulcers for many years.

The purpose of this study was to evaluate the effects of HBOT on hematological indices and biochemical parameters in patients with diabetic foot.

## 2. Methods

### 2.1. Study population

The study was conducted in the patients who applied to the Yunus Emre State Hospital HBOT Center between September 1, 2016 and December 31, 2020, and had received HBOT with a diagnosis of a diabetic foot.

### 2.2. Inclusion and exclusion criteria

All male patients who were treated with a diagnosis of diabetic wound in HBOT clinic during the study period were included. Pre- and post- treatment hematological indices and biochemical parameters in patient files were recorded.

Patient files did not include blood assays which had been taken before and after HBOT were excluded from the study.

### 2.3. Diabetic patient management

All diabetic patients who applied to HBOT, counseled to internal medicine clinic for total clinical evaluation and treatment plan. All patients were provided diabetic patient education by trained health professionals (nurse) and dietitian calculated the total caloric requirements and translated them into patients’ diet. All patients’ blood glucose levels controlled and regulated oral antidiabetics, insulin, and/or both. Comorbidities were also taken into consideration and counseled to appropriate surgeon or specialist. All patients evaluated daily bases and diabetic wound care executed. Management of patients with diabetic wounds were evaluated by the board of diabetic foot.

### 2.4. Rational of hyperbaric oxygen therapy in diabetic foot

Multiplace Hyperbaric Oxygen Chamber produced by Hipertech Company; Türkiye were used in our center. HBOT performed in chamber pressurized with compressed air (2.4 ATA) and each session lasted for 120-minute. Every 25 minutes of 100% O_2_ application is followed by a 5-minute mask-off cabin air (normoxic) inhalation period. As a regulation in our country, hyperbaric oxygen therapy is performed between 20-60 sessions in 1 year period according to course of the wound and the clinical outcome. In case of major and minor amputation or new wounds on different extremities, maximum number of sessions may increase.

### 2.5. Limitations of the study

HBOT is an approved treatment modality adjuvant to the medication and surgical interventions in Diabetic wound patients. Because of obligatory dictation of the legislation and ethical considerations there were no patients who were treated without HBOT in diabetic wound indication in our clinic. A control group from patients with other HBOT indications might have been constituted but this control group was not ideal or identical because DM is a metabolic disease affecting human physiology especially blood and leads to diabetic vasculopathy, diabetic neuropathy and consequently results in diabetic wound (foot) which was the subject of this study. So, above mentioned reasons there were no control group in this study.

Because of uneven groups’ size, non-normal distribution and a small number of female patients, only male patients were included in this study.

### 2.6. Ethical principles and permissions

All patients were informed about HBOT, and the consent was taken. All stages of the study were carried out according to the principles of the Helsinki Declaration. The study was approved by Gulhane Scientific Research Ethics Committee of University of Health Sciences (2021/59).

### 2.7. Statistical analysis

The data were compiled in the Microsoft Excel program and the IBM SPSS package program was used for statistical analysis. The value of *P* = .05 or lower was accepted as statistically significant. Descriptive statistics of study were provided as frequency, percentage, mean and standard deviation. The distribution of groups was evaluated by the Kolmogorov-Smirnov test. Pre - Post treatment analyses were performed with the “Paired T Test” and “Wilcoxon Signed Ranks Test” Because of non-normal distribution of the parameters Spearman Test was used for correlation analyses.

## 3. Results

Twenty three of the 126 patient files did not include laboratory assays were excluded from the study.103 male patients’ files were analyzed within the scope of the study. Study groups’ parameters of age, total number of the HBOT sessions, elapsed time between pre/post blood tests sampling and the treatments were presented in Table 1.

**Table 1:** Table of the parameters of age, total hyperbaric oxygen therapy number, elapsed time between pre/post blood tests sampling and treatments.

Variable	Patients	
	N	Mean
Age (yr)	103	62.05 ± 10.93
Total number of sessions	103	17.81 ± 9.07
Elapsed time to first hyperbaric oxygen therapy session (d)	103	5.11 ± 4.16
Elapsed time after last hyperbaric oxygen therapy session (d)	103	4.54 ± 5.59

Effects of HBOT on blood parameters investigated via correlation analyses. First before values subtracted from after HBOT values to find delta changes. Two parameters reached statistically significancy with negative low correlations. Correlations found between number of HBOT sessions and MCH and Blood Urea Nitrogen (BUN) were *P* = .037, r = −0.207; *P* = .037, r = −0.222, respectively.

WBC, NEU, MON, Platelet Count (PLT), and PTC parameters were found to be decreased (*P* < .05), and an increase in LYM, EOS, MCHC, and RDW parameters were detected (*P* < .05) after the treatments (Table 2). Among biochemical parameters, Glu, C-Reactive Protein (CRP), direct bilirubin, and TP parameters decreased (*P* < .05) and an increase in UA was detected (*P* < .05) after the treatments (Table 3).

**Table 2:** Comparison of the hematological indices results of participants before and after hyperbaric oxygen therapy.

Parameter	All patients	Before hyperbaric oxygen therapy	After hyperbaric oxygen therapy	*P* value
		Mean	Mean	
WBC, ×10^3^/μL	101	10.50 ± 4.07	8.61 ± 2.97	.000†
NEU, ×10^3^/μL	102	7.46 ± 3.77	5.34 ± 2.75	.000†
LYM, ×10^3^/μL	102	1.98 ± 0.63	2.16 ± 0.68	.003†
MON, ×10^3^/μL	102	0.82 ± 0.34	0.71 ± 0.24	.001†
EOS, ×10^3^/μL	102	0.24 ± 0.20	0.33 ± 0.28	.000†
BAS, ×10^3^/μL	102	0.06 ± 0.08	0.07 ± 0.09	.112†
HGB, g/dL	102	12.13 ± 1.75	12.04 ± 1.78	.200*
HTC, %	102	36.74 ± 5.09	36.22 ± 5.23	.200*
RBC, ×10^6^/μL	102	4.39 ± 0.61	4.38 ± 0.63	.200*
MCV, fL	102	83.86 ± 5.49	82.85 ± 5.02	.200*
MCH, pg	102	27.67 ± 2.07	27.55 ± 1.99	.267*
MCHC, pg	102	32.98 ± 1.22	33.25 ± 1.15	.011†
RDW, %	102	14.17 ± 2.56	14.60 ± 2.10	.000†
PLT,/μL	102	331509.80 ± 103841.15	279686.27 ± 107099.47	.000†
MPV, fL	102	10.12 ± 0.89	10.25 ± 0.91	.059†
PTC, %	102	0.33 ± 0.09	0.28 ± 0.10	.000†
PDW, %	102	8.60 ± 2.21	11.74 ± 2.10	.203†

BAS = Basophils, EOS = Eosinophils, HGB = Hemoglobin, HTC = Hematocrit, LYM = lymphocytes, MCH = Mean Corpuscular Hemoglobin, MCHC = Mean Corpuscular Hemoglobin Concentration, MCV = Mean Corpuscular Volume, MON = Monocytes, MPV = Mean Platelet Volume, NEU = Neutrophils, PDW = Platelet Distribution Width, PLT = Platelet Count, PTC = Plateletcrit, RBC = Red Blood Cell Count, RDW = Red Cell Distribution Width, WBC = White Blood Cell Count.

* *P* < .05.

† *P* < .05.

**Table 3:** Comparison of biochemical parameters results of participants before and after hyperbaric oxygen therapy.

Parameter	All patients	Before hyperbaric oxygen therapy	After hyperbaric oxygen therapy	*P* value
		Mean	Mean	
AST, U/L	85	17.52 ± 8.63	16.24 ± 6.05	0.729†
ALT, U/L	91	11.46 ± 11.09	19.73 ± 13.26	0.725†
ALP, U/L	26	101.54 ± 30.37	93.77 ± 30.93	0.284*
GGT, U/L	19	32.21 ± 17.67	31.05 ± 33.17	0.091†
LDH, U/L	3	297.67 ± 253.10	183.67 ± 73.15	0.593†
LDL, mg/dL	3	104.33 ± 18.58	120.67 ± 28.57	0.109†
HDL, mg/dL	4	39.68 ± 14.35	44.78 ± 7.57	0.465†
Cho, mg/dL	6	171.17 ± 34.68	195.83 ± 55.35	0.149*
TG, mg/dL	15	163.47 ± 83.39	199.00 ± 135.18	0.820†
CRP, mg/dL	70	59.46 ± 70.07	18.61 ± 26.76	0.000†
T-Bil, mg/dL	28	0.63 ± 0.34	0.60 ± 0.33	0.111†
D-Bil, mg/dL	17	0.23 ± 0.17	0.19 ± 0.12	0.033†
BUN, mg/dL	89	25.83 ± 15.13	25.43 ± 11.36	0.113†
Cr, mg/dL	91	1.21 ± 0.96	1.11 ± 0.49	0.589†
UA, mg/dL	50	5.44 ± 1.89	5.90 ± 1.43	0.035*
Alb, g/dL	45	3.48 ± 0.60	3.59 ± 0.62	0.246*
TP, g/dL	23	6.94 ± 0.77	6.63 ± 0.70	0.050†
HbA1C, %	6	8.30 ± 2.75	7.67 ± 1.66	0.358*
Glu, mg/dL	84	206.12 ± 108.24	145.50 ± 70.32	0.000†
ESR, mm/h	13	61.00 ± 44.33	50.69 ± 31.67	0.220*

Alb = Albumin, ALP = Alkaline Phosphatase, ALT = Alanine Aminotransferase, AST = Aspartate Aminotransferase, BUN = Blood Urea Nitrogen, Cho = Total Cholesterol, Cr = Creatinine, CRP = C-Reactive Protein, D-Bil = Direct Bilirubin, ESR = Erythrocyte Sedimentation Rate, GGT = Gamma-Glutamyl Transferase, Glu = Blood Glucose, HbA1C = Glycosylated Hemoglobin, HDL = High-Density Lipoprotein, LDH = Lactate Dehydrogenase, LDL = Low-Density Lipoprotein, T-Bil = Total Bilirubin, TG = Triglycerides, TP = Total Protein, UA = Uric Acid.

* *P* < .05.

† *P* < .05.

## 4. Discussion

This study was conducted to evaluate the effects of HBOT on hematological indices and biochemical parameters in patients with diabetic foot. Erythrocyte Sedimentation Rate (ESR), CRP, WBC, NEU and Glu were found to be decreased after HBOT. Before and after treatment results of lipid profile, liver enzymes and kidney functions’ parameters were similar.

DM is a common metabolic disease with significant morbidity and mortality that causes systemic and chronic complications.^[9]^ In International Diabetes Federation 2019 Diabetes Report, 463 million people aged 20 to 79 have DM worldwide, and 760 billion dollars were spent globally for diabetic health expenses in 2019. The International Diabetes Federation predicts that 578 million adults will have diabetes by 2030, and 700 million persons will be affected by 2045. Due to the inflammatory environment in DM causes deterioration in connective tissue metabolism by increasing the oxidative damage to the components of the cell.^[10]^ DM also interferes with the metabolism of cells and tissues. NEU derived cytotoxic enzymes, free radicals and inflammatory mediators prolong the inflammatory stages of the diabetic wounds, slowing down wound healing.^[10]^

HBOT increases the amount of dissolved oxygen in the blood, creating a pressure gradient that increases the diffusion of the oxygen into the tissues. HBOT can increase angiogenesis and fibroblast proliferation by increasing vascular endothelial growth factor and fibroblast growth factor expression. In addition, the resulting hyperoxia can reduce tissue edema by causing vasoconstriction. HBOT suppresses inflammation by decreasing the expression of pro-inflammatory cytokines and boosting the ability of leukocytes to destroy microorganisms.^[6–8]^ Published studies showed that HBOT also lowered major amputation rates in diabetic foot infections.^[11,12]^ Because of these positive effects, HBOT is widely used in the treatment of diabetic foot ulcers.^[13]^

Many physiological factors such as age, gender, environmental factors, and comorbid diseases affect hematological indices.^[13]^ As an environmental factor, HBOT may also affect hematological indices. It has been reported that erythropoiesis suppressed in patients after HBOT and hemoglobin and Red Blood Cell Count values reduced.^[13–15]^ Mean Corpuscular Volume (MCV), MCH, and PLT numbers of divers were lower than non-divers.^[14]^ Acute and chronic effects of HBOT on hematological indices were investigated, and it was found that MCV decreased and RDW increased, but detected changes were not statistically significant.^[15]^ In our study, similar to the literature, decrease in MCV values and increase in RDW values were found after HBOT, and increased RDW values were found to be statistically significant. In addition, PLT counts, and PTC values were found to be lower and statistically significant after HBOT in our study. Like published literature, there were negative low correlations between number of HBOT sessions and MCH values in our study. Contrary to the literature, there were not statistically, or clinically significant changes detected between before and after HBOT in Red Blood Cell Count, hemoglobin, and Hematocrit values in our study.

In diabetic wounds, when inflammation is severe, cytokine levels are found to be high. Oxidative stress is increased during inflammation. CRP release from hepatocytes is also increased by pro-inflammatory cytokines.^[16]^ As mentioned earlier HBOT suppresses inflammation, and it has been shown that HBOT decreased blood CRP levels in diabetic rats.^[16]^ It has also been reported that a reduction in serum CRP and ESR values was reported in patients with diabetic foot wounds who received 120 minutes. HBOT for 20 days at a pressure of 2.5 ATA.^[17]^ In our study, in line with the literature, a decrease in inflammatory/infection indicators such as ESR, CRP, WBC, and NEU was detected after HBOT.

In a HBOT (45 minutes at 2.8 ATA and 55 minutes at 2.0 ATA) study, biochemical parameters that were taken before and 1 week after and Triglycerides (TG) levels increased, total cholesterol (Cho), High-Density Lipoprotein, and Low-Density Lipoprotein values did not change, and parameters of liver and kidney function did not change.^[17]^ It was reported that a decrease detected in BUN, UA, TG, Cho, Aspartate Aminotransferase, and Alanine Aminotransferase values in rats receiving 60 minutes. HBOT for 20 days at a pressure of 2.4 ATA in rats with developed metabolic syndrome.^[18]^ Like published literature, there were negative low correlations between number of HBOT sessions and BUN in our study. Contrary to the literature, an increase in UA was detected in our study. When the effects of HBOT on Cho, TG, kidney, and liver functions were examined, no clinically significant result could be reached.

In a recent study, a decrease seen in Glu, insulin, and Homeostatic Model Assessment for Insulin Resistance values was found in rats with developed metabolic syndrome treated with 60 minutes HBOT for 20 days at a pressure of 2.4 ATA.^[19]^ It was also reported that a reduction in serum Glycosylated Hemoglobin (HbA1c) values reported in patients with diabetic foot wounds who received 120 minutes. HBOT for 20 days at a pressure of 2.5 ATA.^[17]^ In our study, in line with the literature, a statistically significant decrease in Glu was detected. Because the changes in HbA1c levels took some time in diabetic patients, there was no statistical significance in terms of a decrease in HbA1c values in our study.

## 5. Conclusion

Adjuvant HBOT improved hematological indices in diabetic wound patients and had a beneficial effect on biochemical parameters, particularly glucose and CRP levels. As a conclusion, adjuvant HBOT alleviate diabetic inflammation and has a beneficial effect on diabetic patient treatment.

## Author contributions

**Conceptualization:** Erdinç Ercan, Bülent Erdoğan, Fatih Özçelik.

**Data curation:** Erdinç Ercan, Gamze Aydin.

**Formal analysis:** Erdinç Ercan, Gamze Aydin.

**Funding acquisition:** Erdinç Ercan.

**Investigation:** Erdinç Ercan, Gamze Aydin.

**Methodology:** Erdinç Ercan, Gamze Aydin, Bülent Erdoğan, Fatih Özçelik.

**Project administration:** Erdinç Ercan, Gamze Aydin, Bülent Erdoğan.

**Resources:** Erdinç Ercan.

**Software:** Erdinç Ercan.

**Supervision:** Erdinç Ercan, Fatih Özçelik.

**Validation:** Erdinç Ercan, Bülent Erdoğan.

**Visualization:** Erdinç Ercan, Bülent Erdoğan.

**Writing – original draft:** Erdinç Ercan, Gamze Aydin, Bülent Erdoğan, Fatih Özçelik.

**Writing – review & editing:** Erdinç Ercan, Gamze Aydin, Bülent Erdoğan, Fatih Özçelik.
